# Approach to Multiple Pulmonary Nodules: A Case Report and Review of Literature

**DOI:** 10.1100/tsw.2011.74

**Published:** 2011-04-05

**Authors:** Farshid Niknam, Jiezhong Chen, Sabar Napaki, Morteza Aghmesheh

**Affiliations:** ^1^ Wollongong Hospital, Wollongong, NSW, Australia; ^2^ Illawarra Health and Medical Research Institute, University of Wollongong, Wollongong, Australia; ^3^ Illawara Cancer Care Centre, Wollongong Hospital, Wollongong, NSW, Australia

**Keywords:** second opinion, chest X-ray, CT scan, videothoracoscopic lung biopsy, atypical epithelial cells

## Abstract

Chest X-ray and CT examinations often find pulmonary nodules that could be malignant or benign. A case is presented and discussed here in order to improve diagnosis and management of pulmonary nodules. A 62-year-old lady was found to have multiple pulmonary nodules by X-ray when she complained of a cough and fever. This was confirmed by a CT scan. Fine needle aspiration (FNA) of one of the lung lesions reported scant atypical epithelial cells that stained positive for TTF-1 and cytokeratin 7, but negative for cytokeratin 20. Thus, it was suspicious for large cell carcinoma. A videothoracoscopic lung biopsy and histopathology were applied and showed a necrotic nodule with surrounding chronic inflammation and macrophage response, with no evidence of malignant cells. Atypical reactive pneumocytes at the periphery of the lesion (an old infarct) were probably equivalent to the atypical cells seen on cytology. This result changed the diagnosis of our patient from a malignant condition to a benign process. Thus, CT and FNA may give a false positive. A second pathological opinion is very useful for the right diagnosis and management, as shown in our case.

## INTRODUCTION

Pulmonary nodules are a frequent incidental finding on chest computed tomography (CT), which may represent primary lung cancer or other malignant or benign lesions. The primary goal of the evaluation of these nodules is to determine whether they are malignant or benign. Although multiple pulmonary nodules commonly represent lung cancer, most pulmonary nodules are benign when they are <1 cm in diameter[1]. Clinical factors such as older age, tobacco smoking, and a current or remote history of malignancy increase the pretest likelihood of malignancy.

This report aims to conceptualize investigations and the patient's clinical scenario (history, physical examination) in order to prevent wrong diagnosis and management. A case report of the incidental finding of multiple pulmonary nodules in our patient was drafted and the relevant literature about differential diagnosis, diagnostic tools, and managements was reviewed.

## CASE SUMMARY

A 62-year-old lady initially presented to her general practitioner with a few weeks history of a productive cough and fever. This was investigated by a plain chest X-ray, which showed a number of vague, scattered, and small nodular densities in both lung fields, suggestive of secondary neoplastic disease. She denied any weight loss or loss of appetite, chest pain, dyspnea, or fatigue. The patient was a current smoker with a history of smoking 50 packs per year. Her past medical history included anxiety, hyperlipidemia, gastroesophageal reflux disease (GERD), and a partial hysterectomy in 1970. Her family history included maternal pancreatic cancer at age 72 and bowel cancer in her brother at age 40. Her current medications were alprazolam, fluoxetine hydrochloride, simvastatin, and paracetamol.

## INVESTIGATIONS

The multiple pulmonary lesions in our patient were initially investigated by routine hematological and biochemical tests, including tumor markers (CEA, CA 19.9, CA 125, CA15.3, and AFP), which were all normal except for marginally elevated CEA. As part of the radiological investigation, she underwent staging CT scans of the chest, abdomen, and pelvis.

The CT scan of her chest showed multiple bilateral nodules throughout the lung parenchyma as listed in Table 1. There was no evidence of hilar or mediastinal lymphadenopathy and the bronchi appeared to be within the normal limits (Fig. 1). The CT scan of the abdomen and pelvis did not show any signs of lymphadenopathy or lesions.

**TABLE 1**
**CT Scan of Nodules in the Lung Parenchyma**

| Lobe | No. of Nodules | Size | Outline | Calcification/Necrosis (Present or Absent) |
|---|---|---|---|---|
| RUL | 8 | Largest: 7.6 mm<br>Smallest: 4.9 mm | Smooth | Absent/absent |
| RML | 4 | Largest: 7.6 mm<br>Smallest: 4.9 mm | Smooth | Absent/absent |
| RLL | 4 | Largest: 6.6 mm<br>Smallest: 3.2 mm | Smooth | Absent/absent |
| LUL | 5 | Largest: 7.7 mm<br>Smallest: 4.9 mm | Smooth | Absent/absent |
| LLL | 1 | 4.3 mm | Smooth | Absent/absent |

The patient investigation was continued by a fine needle aspiration (FNA) of one of the lung lesions, which was reported as scant, atypical epithelial cells that stained positive for TTF-1 and cytokeratin 7, but negative for cytokeratin 20. Thus, it was suspicious for large cell carcinoma. To identify a possible primary site for these “metastatic deposits” in the lung, our patient underwent further investigations including pelvic ultrasound, bilateral breast mammography, gastroscopy, colonoscopy, and bone scans, which were all reported to be normal. Subsequently, she underwent further investigation by positron emission tomography (PET). This showed faint metabolic activity in the right upper lobe of the lung, the bilateral hilar, and on the right side at the base of tongue. The level of uptake in all these sites was more suggestive of a benign etiology. To exclude a primary malignancy in the tongue, she underwent a direct examination by an ear, nose, and throat specialist, which did not reveal any obvious lesions, especially on the right side at the base of the tongue.

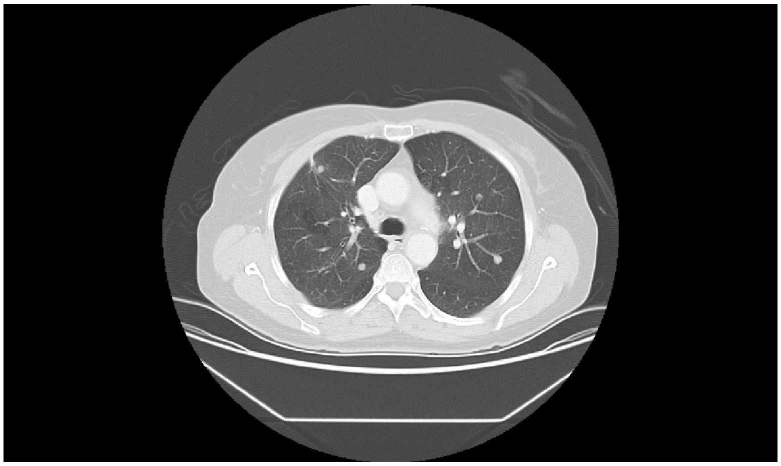

**FIGURE 1**. Multiple nodular densities in the lung fields of a 62-year-old lady. Features are not specific, but highly suggestive of metastases.

Despite the cytology result, the patient remained clinically well; hence, a second opinion was obtained from another pathologist who suggested that the atypical cells may or may not represent a malignancy. The patient was discussed in our lung multidisciplinary team meeting where a thoracoscopic biopsy was suggested. Therefore, our patient underwent an uneventful videothoracoscopic lung biopsy (VTLB) and histopathology, which showed a necrotic nodule with surrounding chronic inflammation and macrophage response, with no evidence of malignant cells. The possibilities of a rheumatoid nodule and infection were considered, but special stains for organisms, including those for mycobacteria, were negative and the presence of ghost outlines of vessels and lung parenchyma in the necrotic focus were more suggestive of an old infarct. Atypical reactive pneumocytes at the periphery of the lesion were probably equivalent to the atypical cells seen by cytological examination (Figs. 2 and 3). The suspicious cells might also have originated from another, more centrally located lesion.

This result changed the diagnosis of our patient from a malignant condition to a benign process. To further investigate the cause of the pulmonary infarction, a transthoracic echocardiogram was conducted to investigate the source. It was reported to be normal. Her vasculitic screenings (ANA, ANCA IF, MPO ANCA, PR3 ANCA, DS DNA, ENA Screen, Cardiolipin Ab, Hepatitis B and C) were all within the normal limits. To date, we were unable to identify the cause for the patient's multiple pulmonary infarcts and she remains clinically stable 12 months after the initial diagnosis of her pulmonary lesions, with no new changes evident on her follow-up chest CT-scan.

## DISCUSSION

The availability and increasing number of chest CT scans in patients with pulmonary complaint cause frequent incidental findings of multiple pulmonary nodules. Multiple pulmonary nodules may represent primary lung cancer or other malignant or benign lesions. In addition, intrapulmonary metastatic tumor may also coexist with nonmalignant lesions, such as hamartomas, lymphoid hyperplasia, and infection[2]. Thus, it is critical to identify whether these nodules are malignant or benign, although most of them are benign[1,2], including infectious granulomas (tuberculosis, histoplasmosis, coccidiodomycosis), sarcoidosis, Wegener's granulomatosis, rheumatoid nodules, benign neoplasms, septic emboli, pulmonary infarct, and Churg Strauss syndrome[2,3,4]. It is important to be able to distinguish whether they are malignant or benign quickly and accurately. It is essential to evaluate clinical factors (such as older age, tobacco smoking, and current or remote history of malignancy), take a proper history, examine, and organize systemic relevant investigations to prove or exclude benign causes.

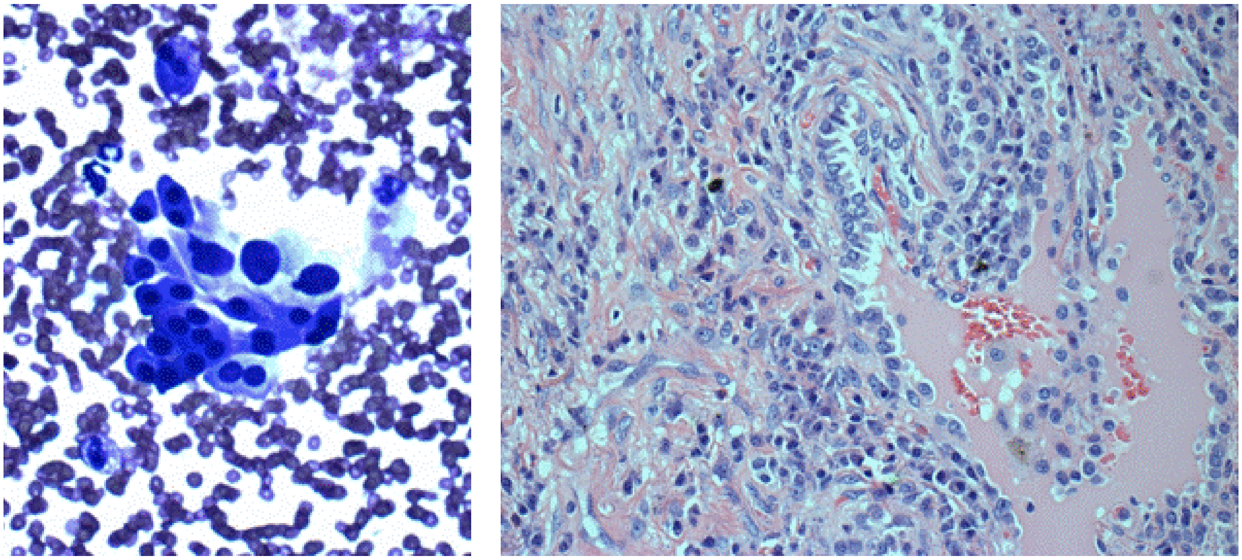

A B

**FIGURE 2.** FNA biopsy (A) and VTLB (B) of the lung lesions in a 62-year-old lady who presented with multiple pulmonary nodules. (A) Atypical cells on cytology suspicious of large cell carcinoma; (B) equivalent cells (pneumocytes) in tissue section.

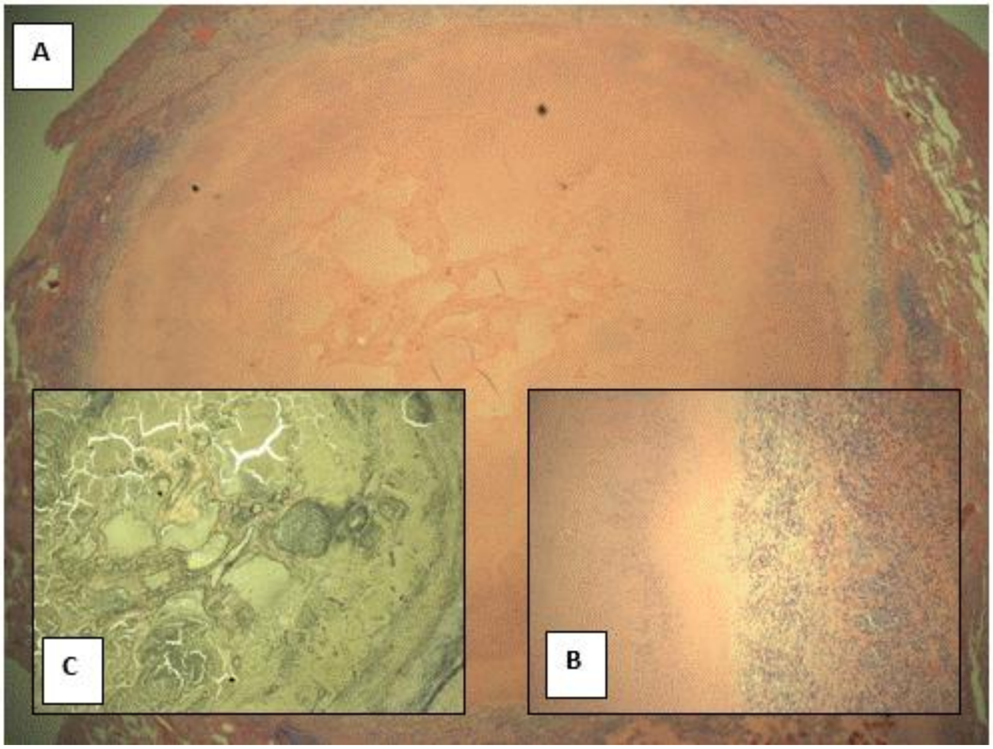

**FIGURE 3.** Histologic features of a lung lesion biopsied via videothoracoscopic procedure in a 62-year-old lady who presented with multiple pulmonary nodules. (A) Necrotic nodule; (B) histiocytic rim of necrotic nodule; (C) retic stain showing vessel outlines.

The chest CT scan and FNA biopsy are the most common, well-established, and important tools for diagnosis and preoperative evaluation of pulmonary nodules[5,6,7]. FNA biopsies make cost-effective contributions to the clinical management of multiple pulmonary nodules by reducing unnecessary operations and increasing agreement between physicians on the need for surgery[5]. These two methods provide a definitive diagnosis in most patients at low cost and with minimal trauma. However, because of the nature of the study and the presentation of cells outside the context of tissue, false-positive or negative results can occur.

FNA can obtain satisfactory diagnostic material in almost 86% of cases. This percentage is reduced if the size of the nodule is <8 mm[8,9]. Tomiyama et al. reported a significant difference in the diagnostic accuracy and sensitivity for the biopsy of small nodules (≤15 mm), as 74% (20 of 27 lesions) and 72%, respectively[10]. However, both PET and FNA biopsies are not as reliable for the diagnosis of lesions <8 mm as they are for larger nodules[8,9]. Some authors challenge this concept by criticizing the FNA biopsy as less sensitive in terms of providing too little tissue for evaluation. Artifacts, inadequate specimens, or inaccurate localization of aspiration are the factors that need to be kept in mind as diagnostic pitfalls in the interpretation of FNA biopsies of pulmonary nodules[6]. Furthermore, due to the small size of the specimens, FNA biopsies often do not provide adequate tissue for morphological examination by a pathologist and this could compromise the interpretation of the results.

In our patient, the initial result of the FNA biopsy was reported as scant, atypical epithelial cells, suspicious for a large cell carcinoma. All paraclinical investigations failed to identify a primary site for the "metastatic" lesions in the lung. This may lead a physician to diagnose metastatic carcinoma of unknown primary and to treat with broad-spectrum chemotherapy to cover all possible primary sites. Adequate tissue and a second pathological opinion need to be considered when there is no correlation between the clinical finding and pathological results.

Numerous studies have addressed the role of second opinions within general, surgical, and pathological consultations, and have reported discrepancy rates ranging from 1.3 to 9.1%, with approximately 6% having major therapeutic significance[11,12]. These could be the result of the rare or esoteric nature of the lesion, limited experience of the pathologist, the increasing use of smaller diagnostic biopsy specimens as the standard of practice in that area, or the requirement for ancillary stains or tests not available in all practice settings. Among the second opinion consults, the pathological opinion is the most important one because changes in the interpretation of the pathology can dramatically change both the prognosis and the treatment. In our case, a second pathological opinion altered both the diagnosis and the management of the patients.

Videothoracoscopic biopsy is a good alternative technique when FNA biopsy fails to obtain adequate tissue and where the diagnosis in uncertain. Thoracoscopic biopsy can cause chest discomfort due to incision, general anesthetics, and pneumothorax (7%), but is safe to use and feasible[13,14]. VTLB has decreased morbidity (4.5 to 23.8%), mortality (0 to 4.5%), and hospital stays (2.6 ± 2.2 to 5.7 ± 2.9) compared to open lung biopsy. On the other hand, the diagnostic yield is approximately 95 to 100%[13].

Although our patient did not present with any previous history of pulmonary symptoms to suggest pulmonary emboli, a VTLB confirmed a pulmonary infarction as the cause of pulmonary lesions. Pulmonary embolism has an incidence of approximately two in every 1000/year in Australia and is associated with considerable morbidity and mortality rates[15]. The true incidence of pulmonary embolism is likely higher because an unknown number of patients with this condition are undiagnosed or misdiagnosed. Unsuspected pulmonary embolism can present with an inpatient prevalence of 4.0% and outpatient prevalence of 0.9%[16]. Clinically unsuspected pulmonary embolism occasionally may be detected in patients undergoing a CT scan of the chest for various reasons. The reported incidence of incidental pulmonary embolism varies from 0.5% with conventional sequential CT scanners to 1.5% with single-detector CT scanners[16,17]. Most pulmonary emboli are multiple and the lower lobes are more commonly involved than the upper lobes[18,19].

## CONCLUSION

Tissue contamination can occur with CT scan–guided aspiration techniques, leading to interpretative errors for the unwary cytopathologist. We strongly believe that interpreting the pathology report in the context of the clinical scenario and considering a second opinion in certain patients will prevent wrong diagnosis and management. Reporting these types of errors will raise the awareness of both the clinician and pathologist in the interpretation of FNA biopsies.
